# *N*-(3-Chloro-1-methyl-1*H*-indazol-5-yl)-4-methylbenzene­sulfonamide

**DOI:** 10.1107/S1600536814001184

**Published:** 2014-01-22

**Authors:** Hakima Chicha, El Mostapha Rakib, Ouafa Amiri, Mohamed Saadi, Lahcen El Ammari

**Affiliations:** a Laboratoire de Chimie Organique et Analytique, Université Sultan Moulay Slimane, Faculté des Sciences et Techniques, Béni-Mellal, BP 523, Morocco; b Laboratoire de Chimie du Solide Appliquée, Faculté des Sciences, Université Mohammed V-Agdal, Avenue Ibn Battouta, BP 1014, Rabat, Morocco

## Abstract

The asymmetric unit of the title compound, C_15_H_14_ClN_3_O_2_S, contains two independent mol­ecules showing different conformations: in one mol­ecule, the indazole ring system makes a dihedral angle of 51.5 (1)° with the benzene ring whereas in the other, the indazole unit is almost perpendicular to the benzene ring [dihedral angle 77.7 (1)°]. In the crystal, the mol­ecules are linked by N—H⋯N and N—H⋯O hydrogen bonds, forming a set of four mol­ecules linked in pairs about an inversion centre.

## Related literature   

For the biological activity of sulfonamides, see: El-Sayed *et al.* (2011 ▶); Mustafa *et al.* (2012 ▶); Scozzafava *et al.* (2003 ▶); Abbassi *et al.* (2012 ▶); Bouissane *et al.* (2006 ▶). For similar compounds see: Abbassi *et al.* (2013 ▶); Chicha *et al.* (2013 ▶).
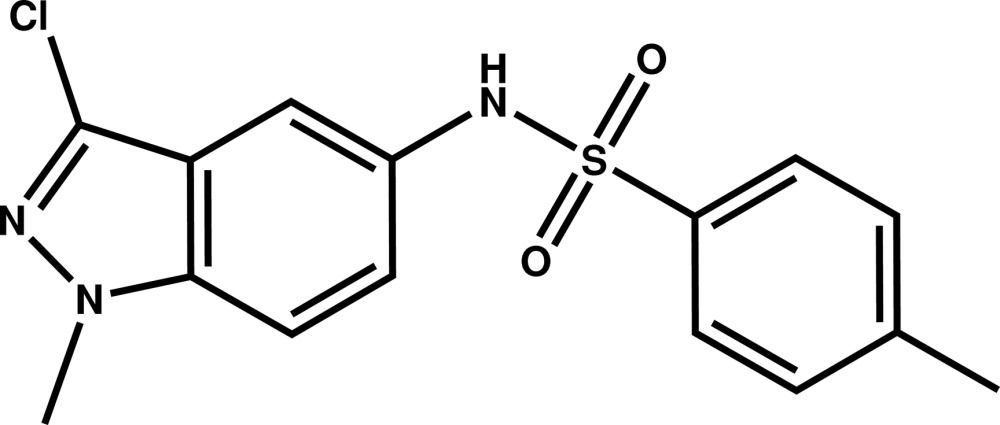


## Experimental   

### 

#### Crystal data  

C_15_H_14_ClN_3_O_2_S
*M* *_r_* = 335.80
Monoclinic, 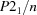

*a* = 8.4580 (13) Å
*b* = 34.920 (6) Å
*c* = 10.8333 (17) Å
β = 97.226 (7)°
*V* = 3174.2 (9) Å^3^
*Z* = 8
Mo *K*α radiation
μ = 0.38 mm^−1^
*T* = 296 K
0.43 × 0.34 × 0.29 mm

#### Data collection  

Bruker X8 APEX Diffractometer
Absorption correction: multi-scan (*SADABS*; Bruker, 2009 ▶) *T*_min_ = 0.693, *T*_max_ = 0.747
24523 measured reflections
7548 independent reflections
5730 reflections with *I* > 2σ(*I*)
*R*_int_ = 0.029

#### Refinement  

*R*[*F* ^2^ > 2σ(*F* ^2^)] = 0.048
*wR*(*F* ^2^) = 0.137
*S* = 1.04
7548 reflections
398 parameters
H-atom parameters constrained
Δρ_max_ = 0.36 e Å^−3^
Δρ_min_ = −0.31 e Å^−3^

### 

Data collection: *APEX2* (Bruker, 2009 ▶); cell refinement: *SAINT* (Bruker, 2009 ▶); data reduction: *SAINT*; program(s) used to solve structure: *SHELXS97* (Sheldrick, 2008 ▶); program(s) used to refine structure: *SHELXL97* (Sheldrick, 2008 ▶); molecular graphics: *ORTEP-3 for Windows* (Farrugia, 2012 ▶); software used to prepare material for publication: *PLATON* (Spek, 2009 ▶) and *publCIF* (Westrip, 2010 ▶).

## Supplementary Material

Crystal structure: contains datablock(s) I. DOI: 10.1107/S1600536814001184/rz5102sup1.cif

Structure factors: contains datablock(s) I. DOI: 10.1107/S1600536814001184/rz5102Isup2.hkl

Supporting information file. DOI: 10.1107/S1600536814001184/rz5102Isup3.cml

Additional supporting information:  crystallographic information; 3D view; checkCIF report

## Figures and Tables

**Table 1:** Hydrogen-bond geometry (Å, °)

*D*—H⋯*A*	*D*—H	H⋯*A*	*D*⋯*A*	*D*—H⋯*A*
N3—H3*N*⋯N5^i^	0.80	2.16	2.952 (3)	167
N6—H6*N*⋯O3^ii^	0.81	2.28	3.022 (2)	152

Symmetry codes: (i) 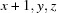; (ii) 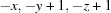.

**Fig. 1.**
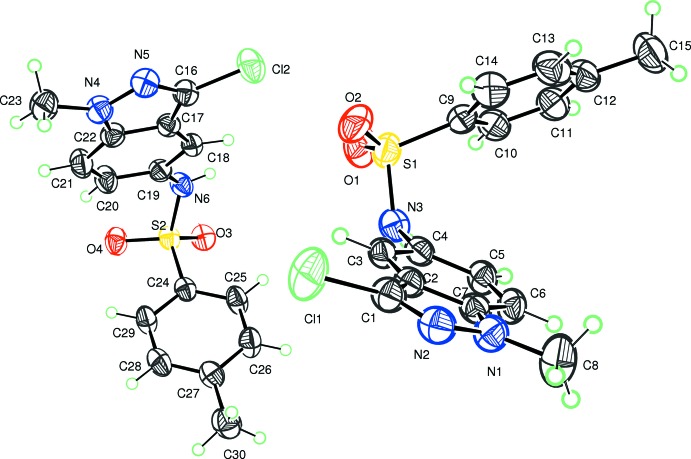
The two independent molecules building the asymmetric unit of the title compound. Displacement ellipsoids are drawn at the 50% probability level. H atoms are represented as small circles.

**Fig. 2.**
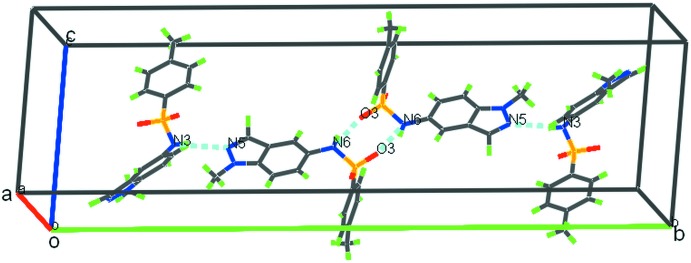
A set of four molecules of the title compound interconnected by hydrogen bonds (dashed line) and linked in pairs by an inversion centre.

## supplementary crystallographic information

### 1. Comment

Sulfonamides are an important class of compounds which are widely used in the design of diverse classes of drug candidates (El-Sayed *et al.*, 2011; Mustafa *et al.*, 2012; Scozzafava *et al.*, 2003). Recently, some *N*-[7(6)-indazolyl]arylsulfonamides prepared by our research group showed important antiproliferative activity against some human and murine cell lines (Abbassi *et al.*, 2012; Bouissane *et al.*, 2006). The present work is a continuation of the investigation of the sulfonamides derivatives published recently by our team (Abbassi *et al.*, 2013; Chicha *et al.*, 2013).

Each of the two independent molecules of the title compound is built up from fused five- and six-membered rings linked to a methylbenzenesulfonamide group as shown in Fig. 1. The molecules show different conformations. In the first molecule, the indazole ring system (N1/N2/C1–C7) makes a dihedral angle of 51.5 (1)° with the plane through the atoms forming the benzene ring (C9–C14). On the other hand, in the second molecule, the fused five- and six-membered ring system (N4/N5/C16–C22) is almost perpendicular to the benzene plane (C24–C29), as indicated by the dihedral angle of 77.7 (1)°. In the crystal, the molecules are interconnected by N3–H3N···N5 and N6–H6N···O3 hydrogen bonds (Table 1) in the way to form a set of four molecules linked in pairs by the inversion centre as shown in Fig. 2.

### 2. Experimental

A mixture of 3-chloro-1-methyl-5-nitroindazole (1.22 mmol) and anhydrous SnCl_2_ (1.1 g, 6.1 mmol) in 25 ml of absolute ethanol was heated at 60 °C for 5 h. After reduction, the starting material disappeared, and the solution was allowed to cool down. The pH was made slightly basic (pH 7–8) by addition of 5% aqueous potassium bicarbonate before extraction with ethyl acetate. The organic phase was washed with brine and dried over magnesium sulfate. The solvent was removed to afford the amine, which was immediately dissolved in pyridine (5 ml) and then reacted with 4-methylbenzenesulfonyl chloride (1.25 mmol) at room temperature for 24 h. After the reaction mixture was concentrated *in vacuo*, the resulting residue was purified by flash chromatography (eluted with ethyl acetate/hexane 2:8 *v*/). The title compound was recrystallized from ethanol, at room temperature, giving colourless crystals (m.p. 397 K, yield: 56%).

### 3. Refinement

All H atoms were located in a difference Fourier map and treated as riding with N—H = 0.89 Å, C–H = 0.93–0.96 Å, and with *U*_iso_(H) = 1.2 *U*_eq_(C, N) or 1.5 *U*_eq_(C) for methyl H atoms. Two outliers (0 1 1, 0 2 1) were omitted in the last cycles of refinement.

### Crystal data

**Table:**

C_15_H_14_ClN_3_O_2_S	*F*(000) = 1392
*M**_r_* = 335.80	*D*_x_ = 1.405 Mg m^−^^3^
Monoclinic, *P*2_1_/*n*	Mo *K*α radiation, λ = 0.71073 Å
Hall symbol: -P 2yn	Cell parameters from 7548 reflections
*a* = 8.4580 (13) Å	θ = 1.2–27.9°
*b* = 34.920 (6) Å	µ = 0.38 mm^−^^1^
*c* = 10.8333 (17) Å	*T* = 296 K
β = 97.226 (7)°	Block, colourless
*V* = 3174.2 (9) Å^3^	0.43 × 0.34 × 0.29 mm
*Z* = 8	

### Data collection

**Table:**

Bruker X8 APEX Diffractometer	7548 independent reflections
Radiation source: fine-focus sealed tube	5730 reflections with *I* > 2σ(*I*)
Graphite monochromator	*R*_int_ = 0.029
φ and ω scans	θ_max_ = 27.9°, θ_min_ = 1.2°
Absorption correction: multi-scan (*SADABS*; Bruker, 2009)	*h* = −11→11
*T*_min_ = 0.693, *T*_max_ = 0.747	*k* = −45→45
24523 measured reflections	*l* = −14→14

### Refinement

**Table:**

Refinement on *F*^2^	Primary atom site location: structure-invariant direct methods
Least-squares matrix: full	Secondary atom site location: difference Fourier map
*R*[*F*^2^ > 2σ(*F*^2^)] = 0.048	Hydrogen site location: inferred from neighbouring sites
*wR*(*F*^2^) = 0.137	H-atom parameters constrained
*S* = 1.04	*w* = 1/[σ^2^(*F*_o_^2^) + (0.066*P*)^2^ + 1.0641*P*] where *P* = (*F*_o_^2^ + 2*F*_c_^2^)/3
7548 reflections	(Δ/σ)_max_ = 0.001
398 parameters	Δρ_max_ = 0.36 e Å^−^^3^
0 restraints	Δρ_min_ = −0.31 e Å^−^^3^

### Special details

**Table:**

Geometry. All e.s.d.'s (except the e.s.d. in the dihedral angle between two l.s. planes) are estimated using the full covariance matrix. The cell e.s.d.'s are taken into account individually in the estimation of e.s.d.'s in distances, angles and torsion angles; correlations between e.s.d.'s in cell parameters are only used when they are defined by crystal symmetry. An approximate (isotropic) treatment of cell e.s.d.'s is used for estimating e.s.d.'s involving l.s. planes.
Refinement. Refinement of *F*^2^ against all reflections. The weighted *R*-factor *wR* and goodness of fit *S* are based on *F*^2^, conventional *R*-factors *R* are based on *F*, with *F* set to zero for negative *F*^2^. The threshold expression of *F*^2^ > σ(*F*^2^) is used only for calculating *R*-factors(gt) *etc*. and is not relevant to the choice of reflections for refinement. *R*-factors based on *F*^2^ are statistically about twice as large as those based on *F*, and *R*- factors based on all data will be even larger.

### Fractional atomic coordinates and isotropic or equivalent isotropic displacement parameters (Å^2^)

**Table:**

	*x*	*y*	*z*	*U*_iso_*/*U*_eq_	
C1	1.0746 (3)	0.91347 (6)	0.79881 (19)	0.0504 (5)	
C2	1.0407 (2)	0.87989 (6)	0.72723 (17)	0.0419 (4)	
C3	0.9081 (2)	0.85660 (6)	0.69368 (18)	0.0434 (4)	
H3	0.8109	0.8619	0.7216	0.052*	
C4	0.9252 (2)	0.82564 (6)	0.61843 (18)	0.0423 (4)	
C5	1.0743 (3)	0.81730 (6)	0.57778 (19)	0.0486 (5)	
H5	1.0831	0.7959	0.5278	0.058*	
C6	1.2054 (3)	0.83968 (6)	0.60973 (19)	0.0495 (5)	
H6	1.3029	0.8339	0.5830	0.059*	
C7	1.1868 (2)	0.87174 (6)	0.68471 (17)	0.0420 (4)	
C8	1.4506 (3)	0.90628 (9)	0.7010 (3)	0.0770 (8)	
H8A	1.4792	0.8864	0.6466	0.115*	
H8B	1.5234	0.9061	0.7765	0.115*	
H8C	1.4556	0.9307	0.6608	0.115*	
C9	0.7224 (2)	0.81651 (6)	0.3369 (2)	0.0480 (5)	
C10	0.7436 (3)	0.78368 (7)	0.2700 (2)	0.0555 (5)	
H10	0.7121	0.7601	0.2983	0.067*	
C11	0.8113 (3)	0.78596 (8)	0.1618 (2)	0.0630 (6)	
H11	0.8253	0.7637	0.1170	0.076*	
C12	0.8592 (3)	0.82078 (9)	0.1178 (2)	0.0647 (7)	
C13	0.8353 (3)	0.85328 (8)	0.1854 (3)	0.0699 (7)	
H13	0.8658	0.8769	0.1565	0.084*	
C14	0.7677 (3)	0.85179 (7)	0.2943 (2)	0.0631 (6)	
H14	0.7526	0.8740	0.3386	0.076*	
C15	0.9348 (4)	0.82282 (12)	−0.0009 (3)	0.0958 (11)	
H15A	0.9758	0.8481	−0.0105	0.144*	
H15B	0.8564	0.8170	−0.0704	0.144*	
H15C	1.0203	0.8046	0.0026	0.144*	
C16	−0.0868 (3)	0.68888 (6)	0.5237 (2)	0.0461 (5)	
C17	−0.1129 (2)	0.65302 (6)	0.57713 (18)	0.0400 (4)	
C18	−0.0879 (2)	0.61439 (6)	0.54823 (17)	0.0409 (4)	
H18	−0.0357	0.6077	0.4807	0.049*	
C19	−0.1426 (2)	0.58688 (6)	0.62205 (18)	0.0424 (4)	
C20	−0.2169 (3)	0.59712 (7)	0.7270 (2)	0.0550 (6)	
H20	−0.2535	0.5779	0.7755	0.066*	
C21	−0.2365 (3)	0.63448 (7)	0.7594 (2)	0.0558 (6)	
H21	−0.2825	0.6409	0.8302	0.067*	
C22	−0.1853 (2)	0.66258 (6)	0.68257 (19)	0.0439 (4)	
C23	−0.2682 (3)	0.72545 (7)	0.7723 (2)	0.0621 (6)	
H23A	−0.3477	0.7416	0.7275	0.093*	
H23B	−0.1879	0.7411	0.8181	0.093*	
H23C	−0.3170	0.7095	0.8289	0.093*	
C24	0.1019 (2)	0.52481 (6)	0.76926 (18)	0.0426 (4)	
C25	0.2386 (3)	0.52368 (7)	0.7115 (2)	0.0555 (6)	
H25	0.2331	0.5167	0.6282	0.067*	
C26	0.3831 (3)	0.53297 (7)	0.7785 (2)	0.0590 (6)	
H26	0.4748	0.5321	0.7393	0.071*	
C27	0.3950 (3)	0.54353 (6)	0.9019 (2)	0.0512 (5)	
C28	0.2566 (3)	0.54467 (8)	0.9573 (2)	0.0630 (6)	
H28	0.2620	0.5518	1.0405	0.076*	
C29	0.1113 (3)	0.53550 (7)	0.8926 (2)	0.0581 (6)	
H29	0.0196	0.5365	0.9318	0.070*	
C30	0.5535 (3)	0.55361 (9)	0.9739 (2)	0.0703 (7)	
H30A	0.5504	0.5487	1.0607	0.105*	
H30B	0.5756	0.5802	0.9622	0.105*	
H30C	0.6356	0.5383	0.9447	0.105*	
N1	1.2903 (2)	0.89975 (5)	0.72974 (16)	0.0508 (4)	
N2	1.2212 (2)	0.92559 (5)	0.79962 (17)	0.0536 (5)	
N3	0.7936 (2)	0.80055 (5)	0.58368 (17)	0.0510 (4)	
H3N	0.8174	0.7786	0.5734	0.061*	
N4	−0.1959 (2)	0.70164 (5)	0.68515 (17)	0.0507 (4)	
N5	−0.1351 (2)	0.71778 (5)	0.58704 (18)	0.0511 (4)	
N6	−0.1321 (2)	0.54756 (5)	0.58771 (16)	0.0481 (4)	
H6N	−0.1053	0.5437	0.5194	0.048 (6)*	
O1	0.5382 (2)	0.78159 (7)	0.47439 (19)	0.0876 (7)	
O2	0.5960 (2)	0.85057 (6)	0.51171 (18)	0.0796 (6)	
O3	−0.06493 (19)	0.48003 (4)	0.60889 (14)	0.0518 (4)	
O4	−0.19633 (19)	0.51112 (5)	0.77251 (15)	0.0572 (4)	
S1	0.64646 (7)	0.81318 (2)	0.47990 (6)	0.05832 (17)	
S2	−0.08315 (6)	0.512747 (14)	0.68561 (5)	0.04431 (14)	
Cl1	0.94290 (10)	0.93782 (2)	0.87784 (8)	0.0867 (3)	
Cl2	−0.00273 (9)	0.697034 (19)	0.39037 (6)	0.06916 (19)	

### Atomic displacement parameters (Å^2^)

**Table:**

	*U*^11^	*U*^22^	*U*^33^	*U*^12^	*U*^13^	*U*^23^
C1	0.0641 (14)	0.0440 (11)	0.0436 (11)	0.0153 (10)	0.0092 (10)	−0.0055 (9)
C2	0.0534 (11)	0.0389 (10)	0.0338 (9)	0.0118 (9)	0.0069 (8)	0.0006 (8)
C3	0.0489 (11)	0.0449 (11)	0.0380 (10)	0.0096 (9)	0.0113 (8)	0.0027 (8)
C4	0.0500 (11)	0.0416 (10)	0.0357 (9)	0.0032 (9)	0.0071 (8)	0.0031 (8)
C5	0.0575 (12)	0.0465 (12)	0.0435 (11)	0.0050 (10)	0.0131 (9)	−0.0104 (9)
C6	0.0505 (11)	0.0533 (13)	0.0468 (11)	0.0059 (10)	0.0145 (9)	−0.0097 (10)
C7	0.0495 (11)	0.0432 (11)	0.0334 (9)	0.0062 (9)	0.0050 (8)	−0.0019 (8)
C8	0.0678 (16)	0.0822 (19)	0.0854 (19)	−0.0212 (14)	0.0272 (14)	−0.0310 (16)
C9	0.0411 (10)	0.0535 (13)	0.0486 (12)	−0.0007 (9)	0.0028 (9)	0.0009 (10)
C10	0.0622 (13)	0.0516 (13)	0.0525 (13)	−0.0028 (11)	0.0058 (10)	0.0013 (10)
C11	0.0679 (15)	0.0713 (17)	0.0494 (13)	0.0015 (13)	0.0058 (11)	−0.0075 (12)
C12	0.0559 (13)	0.092 (2)	0.0455 (12)	−0.0060 (13)	0.0021 (10)	0.0076 (13)
C13	0.0734 (17)	0.0663 (17)	0.0688 (16)	−0.0111 (14)	0.0046 (13)	0.0183 (14)
C14	0.0682 (15)	0.0523 (14)	0.0684 (16)	−0.0010 (12)	0.0066 (12)	−0.0025 (12)
C15	0.084 (2)	0.152 (3)	0.0528 (15)	−0.015 (2)	0.0146 (14)	0.0132 (19)
C16	0.0468 (11)	0.0431 (11)	0.0490 (11)	−0.0042 (9)	0.0083 (9)	0.0018 (9)
C17	0.0390 (9)	0.0407 (10)	0.0398 (10)	−0.0011 (8)	0.0036 (8)	0.0007 (8)
C18	0.0430 (10)	0.0432 (11)	0.0369 (9)	0.0013 (8)	0.0071 (8)	−0.0018 (8)
C19	0.0483 (11)	0.0366 (10)	0.0425 (10)	0.0043 (8)	0.0060 (8)	0.0009 (8)
C20	0.0732 (15)	0.0443 (12)	0.0520 (12)	0.0069 (11)	0.0256 (11)	0.0078 (10)
C21	0.0726 (15)	0.0502 (13)	0.0488 (12)	0.0104 (11)	0.0241 (11)	0.0028 (10)
C22	0.0454 (10)	0.0399 (11)	0.0462 (11)	0.0052 (8)	0.0051 (8)	−0.0023 (8)
C23	0.0732 (16)	0.0530 (14)	0.0616 (14)	0.0132 (12)	0.0150 (12)	−0.0079 (11)
C24	0.0545 (11)	0.0334 (10)	0.0416 (10)	0.0005 (9)	0.0123 (9)	0.0024 (8)
C25	0.0614 (13)	0.0672 (15)	0.0401 (11)	−0.0117 (11)	0.0156 (10)	−0.0138 (10)
C26	0.0577 (13)	0.0688 (16)	0.0536 (13)	−0.0105 (12)	0.0191 (10)	−0.0163 (11)
C27	0.0633 (13)	0.0449 (12)	0.0459 (11)	−0.0046 (10)	0.0091 (10)	−0.0046 (9)
C28	0.0758 (16)	0.0758 (17)	0.0386 (11)	0.0001 (13)	0.0127 (11)	−0.0094 (11)
C29	0.0641 (14)	0.0699 (16)	0.0439 (12)	0.0031 (12)	0.0214 (11)	−0.0026 (11)
C30	0.0760 (17)	0.0789 (18)	0.0556 (14)	−0.0116 (14)	0.0068 (12)	−0.0109 (13)
N1	0.0536 (10)	0.0516 (11)	0.0473 (10)	0.0013 (8)	0.0073 (8)	−0.0106 (8)
N2	0.0667 (12)	0.0454 (10)	0.0483 (10)	0.0062 (9)	0.0057 (9)	−0.0097 (8)
N3	0.0577 (11)	0.0457 (10)	0.0506 (10)	−0.0035 (8)	0.0112 (8)	−0.0002 (8)
N4	0.0585 (11)	0.0412 (10)	0.0533 (10)	0.0038 (8)	0.0107 (8)	−0.0025 (8)
N5	0.0547 (10)	0.0414 (10)	0.0574 (11)	−0.0030 (8)	0.0083 (8)	−0.0002 (8)
N6	0.0656 (11)	0.0393 (9)	0.0410 (9)	0.0040 (8)	0.0126 (8)	0.0009 (7)
O1	0.0602 (11)	0.1231 (18)	0.0819 (13)	−0.0364 (11)	0.0190 (9)	−0.0040 (12)
O2	0.0652 (11)	0.0982 (15)	0.0771 (12)	0.0316 (10)	0.0148 (9)	−0.0131 (11)
O3	0.0635 (9)	0.0352 (7)	0.0582 (9)	−0.0032 (7)	0.0131 (7)	−0.0004 (6)
O4	0.0609 (9)	0.0524 (9)	0.0629 (10)	−0.0027 (7)	0.0257 (8)	0.0083 (7)
S1	0.0431 (3)	0.0763 (4)	0.0573 (3)	−0.0024 (3)	0.0130 (2)	−0.0057 (3)
S2	0.0526 (3)	0.0346 (3)	0.0478 (3)	−0.0019 (2)	0.0143 (2)	0.0037 (2)
Cl1	0.0885 (5)	0.0726 (5)	0.1041 (6)	0.0191 (4)	0.0319 (4)	−0.0360 (4)
Cl2	0.0874 (5)	0.0592 (4)	0.0663 (4)	−0.0082 (3)	0.0308 (3)	0.0107 (3)

### Geometric parameters (Å, º)

**Table:**

C1—N2	1.309 (3)	C18—C19	1.367 (3)
C1—C2	1.415 (3)	C18—H18	0.9300
C1—Cl1	1.713 (2)	C19—C20	1.413 (3)
C2—C3	1.396 (3)	C19—N6	1.428 (3)
C2—C7	1.401 (3)	C20—C21	1.366 (3)
C3—C4	1.373 (3)	C20—H20	0.9300
C3—H3	0.9300	C21—C22	1.390 (3)
C4—C5	1.418 (3)	C21—H21	0.9300
C4—N3	1.429 (3)	C22—N4	1.367 (3)
C5—C6	1.365 (3)	C23—N4	1.450 (3)
C5—H5	0.9300	C23—H23A	0.9600
C6—C7	1.404 (3)	C23—H23B	0.9600
C6—H6	0.9300	C23—H23C	0.9600
C7—N1	1.361 (3)	C24—C29	1.380 (3)
C8—N1	1.447 (3)	C24—C25	1.383 (3)
C8—H8A	0.9600	C24—S2	1.758 (2)
C8—H8B	0.9600	C25—C26	1.379 (3)
C8—H8C	0.9600	C25—H25	0.9300
C9—C10	1.380 (3)	C26—C27	1.379 (3)
C9—C14	1.386 (3)	C26—H26	0.9300
C9—S1	1.754 (2)	C27—C28	1.382 (3)
C10—C11	1.370 (3)	C27—C30	1.505 (3)
C10—H10	0.9300	C28—C29	1.374 (4)
C11—C12	1.385 (4)	C28—H28	0.9300
C11—H11	0.9300	C29—H29	0.9300
C12—C13	1.379 (4)	C30—H30A	0.9600
C12—C15	1.509 (4)	C30—H30B	0.9600
C13—C14	1.376 (4)	C30—H30C	0.9600
C13—H13	0.9300	N1—N2	1.356 (2)
C14—H14	0.9300	N3—S1	1.630 (2)
C15—H15A	0.9600	N3—H3N	0.8039
C15—H15B	0.9600	N4—N5	1.360 (3)
C15—H15C	0.9600	N6—S2	1.6321 (17)
C16—N5	1.314 (3)	N6—H6N	0.8114
C16—C17	1.409 (3)	O1—S1	1.430 (2)
C16—Cl2	1.712 (2)	O2—S1	1.429 (2)
C17—C22	1.403 (3)	O3—S2	1.4325 (15)
C17—C18	1.407 (3)	O4—S2	1.4256 (15)
			
N2—C1—C2	113.42 (18)	C21—C20—H20	119.0
N2—C1—Cl1	120.72 (17)	C19—C20—H20	119.0
C2—C1—Cl1	125.85 (18)	C20—C21—C22	117.6 (2)
C3—C2—C7	120.60 (18)	C20—C21—H21	121.2
C3—C2—C1	136.53 (19)	C22—C21—H21	121.2
C7—C2—C1	102.86 (19)	N4—C22—C21	131.7 (2)
C4—C3—C2	118.20 (18)	N4—C22—C17	106.89 (18)
C4—C3—H3	120.9	C21—C22—C17	121.34 (19)
C2—C3—H3	120.9	N4—C23—H23A	109.5
C3—C4—C5	120.68 (19)	N4—C23—H23B	109.5
C3—C4—N3	120.20 (18)	H23A—C23—H23B	109.5
C5—C4—N3	119.08 (18)	N4—C23—H23C	109.5
C6—C5—C4	122.01 (19)	H23A—C23—H23C	109.5
C6—C5—H5	119.0	H23B—C23—H23C	109.5
C4—C5—H5	119.0	C29—C24—C25	119.7 (2)
C5—C6—C7	117.16 (19)	C29—C24—S2	120.19 (17)
C5—C6—H6	121.4	C25—C24—S2	120.06 (16)
C7—C6—H6	121.4	C26—C25—C24	119.5 (2)
N1—C7—C2	106.86 (17)	C26—C25—H25	120.3
N1—C7—C6	131.81 (19)	C24—C25—H25	120.3
C2—C7—C6	121.33 (19)	C27—C26—C25	121.6 (2)
N1—C8—H8A	109.5	C27—C26—H26	119.2
N1—C8—H8B	109.5	C25—C26—H26	119.2
H8A—C8—H8B	109.5	C26—C27—C28	117.8 (2)
N1—C8—H8C	109.5	C26—C27—C30	121.1 (2)
H8A—C8—H8C	109.5	C28—C27—C30	121.1 (2)
H8B—C8—H8C	109.5	C29—C28—C27	121.6 (2)
C10—C9—C14	120.3 (2)	C29—C28—H28	119.2
C10—C9—S1	119.66 (18)	C27—C28—H28	119.2
C14—C9—S1	120.01 (19)	C28—C29—C24	119.7 (2)
C11—C10—C9	119.8 (2)	C28—C29—H29	120.1
C11—C10—H10	120.1	C24—C29—H29	120.1
C9—C10—H10	120.1	C27—C30—H30A	109.5
C10—C11—C12	121.1 (2)	C27—C30—H30B	109.5
C10—C11—H11	119.4	H30A—C30—H30B	109.5
C12—C11—H11	119.4	C27—C30—H30C	109.5
C13—C12—C11	118.2 (2)	H30A—C30—H30C	109.5
C13—C12—C15	121.3 (3)	H30B—C30—H30C	109.5
C11—C12—C15	120.5 (3)	N2—N1—C7	112.03 (17)
C14—C13—C12	121.9 (3)	N2—N1—C8	119.78 (19)
C14—C13—H13	119.0	C7—N1—C8	127.85 (19)
C12—C13—H13	119.0	C1—N2—N1	104.81 (17)
C13—C14—C9	118.8 (2)	C4—N3—S1	121.17 (15)
C13—C14—H14	120.6	C4—N3—H3N	114.9
C9—C14—H14	120.6	S1—N3—H3N	110.2
C12—C15—H15A	109.5	N5—N4—C22	111.42 (17)
C12—C15—H15B	109.5	N5—N4—C23	120.21 (18)
H15A—C15—H15B	109.5	C22—N4—C23	128.28 (19)
C12—C15—H15C	109.5	C16—N5—N4	105.25 (17)
H15A—C15—H15C	109.5	C19—N6—S2	124.46 (14)
H15B—C15—H15C	109.5	C19—N6—H6N	115.4
N5—C16—C17	113.14 (19)	S2—N6—H6N	113.2
N5—C16—Cl2	120.16 (16)	O2—S1—O1	120.36 (13)
C17—C16—Cl2	126.69 (16)	O2—S1—N3	107.83 (11)
C22—C17—C18	120.18 (18)	O1—S1—N3	104.45 (12)
C22—C17—C16	103.29 (18)	O2—S1—C9	107.82 (12)
C18—C17—C16	136.51 (19)	O1—S1—C9	108.55 (11)
C19—C18—C17	118.18 (18)	N3—S1—C9	107.12 (10)
C19—C18—H18	120.9	O4—S2—O3	118.79 (10)
C17—C18—H18	120.9	O4—S2—N6	108.81 (10)
C18—C19—C20	120.70 (19)	O3—S2—N6	104.71 (9)
C18—C19—N6	119.09 (18)	O4—S2—C24	107.30 (10)
C20—C19—N6	120.11 (18)	O3—S2—C24	109.65 (10)
C21—C20—C19	121.9 (2)	N6—S2—C24	107.03 (10)

### Hydrogen-bond geometry (Å, º)

**Table:**

*D*—H···*A*	*D*—H	H···*A*	*D*···*A*	*D*—H···*A*
N3—H3*N*···N5^i^	0.80	2.16	2.952 (3)	167
N6—H6*N*···O3^ii^	0.81	2.28	3.022 (2)	152

Symmetry codes: (i) *x*+1, *y*, *z*; (ii) −*x*, −*y*+1, −*z*+1.
